# Perinatal complications and socio-economic differences in cerebral palsy in Sweden – a national cohort study

**DOI:** 10.1186/1471-2431-8-49

**Published:** 2008-10-30

**Authors:** Anders Hjern, Kristina Thorngren-Jerneck

**Affiliations:** 1 Centre for Epidemiology, National Board of Health and Welfare, Stockholm, Sweden; 2 Department of CLINTEC, Karolinska Institute, Huddinge, Sweden; 3 Department of Paediatrics, University Hospital, Lund University, Sweden

## Abstract

**Background:**

There is a controversy regarding the existence of a socio-economic gradient for cerebral palsy. Perinatal emergencies and preterm birth increase the risk for the offspring to develop cerebral palsy. The aim of this study was to investigate the association of socio-economic indicators with cerebral palsy (CP) and the role of perinatal health as mediator of this association.

**Methods:**

Register study of a national cohort of 805,543 children born 1987–93, including 1,437 children with cerebral palsy that were identified in hospital discharge data from national registers. Socio-economic indicators of the household were taken from the Census of 1985. Logistic regression and chi-square analyses of linearity were used to test hypotheses.

**Results:**

There was a linear association between the incidence of CP (excluding cases caused by registered injuries or malformations) as well as of major perinatal indicators and the socio-economic status (SES) of the household of the mother (p < 0.001). Children in households with low SES had a higher odds ratio of CP (OR 1.49 [95% C.I. 1.16–1.91]) compared with high SES after adjustment for demographic confounders. This OR decreased to 1.36 (1.05–1.71) after adjustment for perinatal indicators with preterm birth as the most important mediating variable.

**Conclusion:**

This study suggests that there is a continuous socio-economic gradient for CP in Sweden. Further studies in more complete populations of children with cerebral palsy are needed to confirm this. Perinatal complications seem to mediate some of this gradient.

## Background

Many European societies report considerable socio-economic differences in child health [1]. Sweden is no exception, reporting significant socio-economic gradients for major child health problems such as perinatal health, injuries and asthma [2]. Understanding the pathways of the risk factors responsible for these socio-economic differences is essential information for prevention.

Cerebral palsy (CP) is a common cause of severe disability in children. There is conflicting evidence in the literature regarding socio-economic differences in families that give birth to children that develop CP [3]. The only Swedish study up to date reported a slightly higher risk for CP in offspring of mothers with a high socio-economic status (SES) compared with low SES [4]. In contrast studies from the UK [3,5] and Ireland [6] have reported a higher risk of CP in offspring of mothers with a low social class compared with offspring from the highest social class [3].

Preterm birth, low birth weight and perinatal health problems, such as intrapartal emergencies with birth asphyxia, have been linked to the development of CP [7,8]. In this study we used data from Swedish national registers to investigate socio-economic differences in cerebral palsy in seven birth cohorts in Sweden in the presence of perinatal health indicators. Are there socio-economic differences in the incidence of cerebral palsy in Sweden, and if so are these mediated by perinatal risk factors?

## Methods

This study was based on Swedish national registers held by the National Board of Health and Welfare and Statistics Sweden. These registers can be used for statistical reports by government agencies and are also open for researchers after ethical approval by the responsible bodies. This study was approved for two reasons: Firstly, the study was conducted on behalf of the National Board of Health and Welfare, and thus according to the Swedish regulations studies commissioned by the National Board of Health and Welfare does not need an ethical approval. Secondly, all analyses were made on a totally anonymous dataset where all personal identifiers, such as the Swedish national registration number, names or addresses had been ereased.

All children born in Sweden during 1987–93 were identified in the Swedish Medical Birth Register (SMBR) [9]. From this population we excluded children reported to have died during their first year of life according to the National Cause of Death Register and children where the person identification number of the mother was not present in the SMBR, leaving 805,543 children to be included in the study population. The person identification number of the father was added through linkage with the Multi-Generation Register and socio-economic variables of the parental household through linkage to the census of 1985.

The family situation at the first visit to the maternal health clinic, the geographic location of the home and the age of the mother at the birth of the child, year of birth, sex and gestational age of the child were identified in the SMBR. The geographical location of the home was in three category domicile variable according to the degree of urbanisation of the community; metropolitan (Stockholm, Göteborg, Malmö), other city, rural. Gestational age was defined in the SMBR according to maternal report of last menstrual period and clinical judgement by the attending paediatrician. Small for gestational age [SGA] was defined as < -2SD according to the growth chart developed by Marsal et al [10]. 'Low Apgar' was defined as an Apgar score below <7 at 5 minutes in the SMBR. Indicators of neonatal distress, preeclampsia, abruptio placentae and preterm rupture of the membranes were created by the diagnostic information to the SMBR recorded by the obstetrician and paediatrician who attended the delivery.

The socio-economic status (SES) and housing situation of the household of the mother and the maternal and paternal country of birth were identified in the Swedish Population and Housing Census in November 1985, thus prior to any gestational health problem involving the children in the study. Country of birth of the parents was categorised as Swedish, European, non-European and mixed (=one Swedish-born and one foreign-born parent). If one parent was born in Europe outside of Sweden and the other in a non-European country the category of the mother was used to categorise the child. SES was defined according to a classification used by Statistics Sweden, which is based on occupation but also takes educational level of occupation, type of production and position at work of the head of the household into account [11]. Social welfare benefits received by the household of the mother were added through linkage to the Total Enumeration Income Survey for 1990. The highest completed education of the mother in the Swedish Register of Education of 1990 and categorised by number of years in Low (0–11 years), Intermediate (12–14 years) and High (15+ years).

### Cerebral palsy

CP in this study was defined as having been discharged from a hospital after one year of age with CP as a main or contributory diagnosis according to the Swedish Hospital Discharge Register [12] of 1988–2002. This register includes 99% of over-night stays in Swedish hospitals, but not hospitalisations in day care only [12]. We used the CP codes in the 9^th ^revision of the WHO International Classification (ICD-9) [13] during 1987–96 (343) and the 10^th ^revision of the WHO International Classification of Diseases (ICD-10) [14] during 1997–2002 (G80) to identify these discharges. Children thus identified as having CP with a history of a severe traumatic head injury in the National Patient Discharge Register, a malformation syndrome in the central nervous system and/or a chromosomal aberration registered in the SMBR were identified and analysed separately since these cases might have a particular and disparate pattern of risk factors [15].

### Statistical methods

Chi-square tests of linearity were used to examine associations of SES and domicile with the outcome variables. Multivariate analyses were conducted using unconditional logistic regression with CP not caused by severe head injury or malformation as the outcome variable. 689 individuals with an extremely high or extremely low birth weight in relation to their gestational age and/or length were excluded from the multivariate analysis as probable coding errors in the register.

Year of birth was entered as a continuous variable in the regression models in accordance with a continuously decreasing secular trend. A summarised "asphyxia" variable was created with children having at least one of three perinatal risk factors (low apgar, neonatal distress, abruptio placentae). The six category SES variable (Table 1) was summarised to a four category variable in the regression model; unclassified, low (I+II), moderate (III+IV) and high (V). Socio-demographic and perinatal variables were entered as dichotomised category variables (sometimes using dummy variables) into the models.

**Table 1:** Socio-demographic indicators and cerebral palsy (CP) according to hospital data in Swedish children born 1987–93

			Unspecified CP*		All CP	
		N	Cases	1/1 000	Cases	1/1 000
Sex						
	Male	413 234	740	1.79	848	2.05
	Female	392 309	508	1.29	589	1.50
Domicile						
	Metropolitan	256 882	330	1.28	381	1.48
	Other City	396 064	642	1.62	741	1.87
	Rural	152 597	272	1.78	311	2.04
Maternal age at birth of child						
	Missing	12 416	25	2.01	32	2.21
	12–19	9 925	21	2.12	21	2.12
	20–24	182 640	289	1.58	334	1.82
	25–29	300 108	434	1.45	486	1.62
	30–34	205 479	313	1.52	365	1.77
	35+	94 975	166	1.75	199	2.10
SES						
	Unspecified	212 676	335	1.58	392	1.85
	I: Manual labour	189 923	334	1.76	379	2.00
	II: Skilled labor	110 017	177	1.61	198	1.80
	III: White collar 1	101 995	140	1.37	157	1.54
	IV: White collar 2	126 271	189	1.50	222	1.76
	V: White collar 3	64 661	73	1.13	89	1.38
Maternal education						
	Missing	23 966	43	1.79	55	2.29
	Low	146 980	238	1.62	285	1.94
	Moderate	438 087	720	1.64	806	1.84
	High	196 510	247	1.26	291	1.31
Recieved Social Welfare						
	No	747 365	1139	1.52	1304	1.74
	Yes	58 178	109	1.87	133	2.28
Single during pregnancy						
	Missing	62 990	136	2.16	156	2.48
	No	701 548	1035	1.48	1193	1.53
	Yes	41 005	73	1.78	84	2.05
Ethnicity						
	Missing	194	0	0	0	0
	Swedish	651 452	1031	1.58	1177	1.81
	Other European	28 500	36	1.26	48	1.68
	Non-European	39 637	59	1.49	68	1.72
	Mixed	85 760	121	1.41	144	1.68

*Total*		*805 543*	*1248*	*1.55*	*1437*	*1.78*

* Excluding cases with hospital diagnoses that indicates traumatic injuries or malformation syndromes

In the first regression model CP was analysed in relation to SES only. In all other models maternal age, sex and year of birth of the child and the geographic location of the home (residency) were added as demographic confounders. In models 3–5 the three major perinatal variables (gestational age, asphyxia, small for gestational age) were analysed separately in relation to SES while all variables were included in the final model 6. The SPSS software package, version 12.0, was used in all statistical analyses.

## Results

There were 1,437 children discharged from hospital at least once with a diagnosis of CP during 1987–2002, indicating a cumulated hospital incidence of CP of 1.78 per 1000 in the cohorts born 1987–93 during this time period. There were 0.03 children per 1000 with CP associated with severe head injuries, 0.21 per 1000 with CP associated with a malformation syndrome leaving 1.55 per 1000 with an "unspecified" CP, 1.79 in boys and 1.29 in girls.

There was a linear association of unspecified CP with SES (p < 0.001; excluding unclassified SES)(Table 1). There were no significant associations or even tendencies of such a linearity associated with severe head injuries or malformations (data not presented in tables). The incidence of unspecified CP was quite similar in the four categories of parental country of birth with Swedish-born parents having the highest incidence (1.58/1000) and other European the lowest incidence (1.26/1000).

In bivariate analyses of perinatal complications and unspecified CP, preterm birth and low Apgar were associated with a particularly high incidence of cerebral palsy, but small for gestational age, pre-eclampsia, abruptio placentae were also significantly associated with CP (p < 0.001) (Table 2). In all 55% of the children with CP had at least one of these perinatal risk factors; OR 8.0 (7.4–9.8) compared with those having no such risk factor.

**Table 2:** Perinatal indicators and odds ratios of unspecified cerebral palsy (N = 804 854)

		N	Cases	OR (95% C.I.)
Parity	1	334 396	532	1
	2	281 002	410	0.9 (0.7–1.1)
	3	130 463	195	0.9 (0.8 -1.1)
	4+	58 993	111	1.2 (1.0–1.5)
Multiple birth	Yes	18 738	100	3.7 (3.0–4.5)
Gestational age (weeks)	22–28	1 798	107	60.4 (49.2–74.5)
	29–32	6 457	184	27.6 (23.5–32.5)
	33–36	40 148	134	3.1 (2.6–3.7)
	37–41	747 172	799	1
	42–45	9 279	17	1.7 (1.1–2.8)
Small for gestational age	Yes	25 427	133	3.7 (3.1–4.4)
Preeclampsia	Yes	21 329	62	1.9 (1.5–2.5)
Abrubtio placentae	Yes	3 778	60	10.9 (8.4–14.1)
Apgar<7 at 5 min	Yes	7 343	242	27.0 (23.5–31.2)
Neonatal distress	Yes	11 611	221	15.0 (12.9–17.4)

*Total*		*804 854*	*1 248*	

The incidences of extreme preterm birth, small for gestational age and asphyxia were found to increase with decreasing SES (Table 3). The rate of of cerebral palsy in children born preterm (below w 36) in households with low SES (1.13%) was similar to those born preterm in households with high and moderate SES (1.07%), p = 0.4.

**Table 3:** SES and major perinatal risk factors

		Extremely preterm birth (w 24–32)		Asphyxia*		Small for gestational age	
	All	N	Incidence (%)	N	Incidence (%)	N	Incidence (%)
**SES**							
Unclassified	212 457	2 350	1.11	5 324	2.51	7 518	3.54
I	189 781	1 991	1.05	4 626	2.44	6 160	3.25
II	109 934	1 108	1.01	2 486	2.26	3 303	3.00
III	101 906	1 048	1.03	2 629	2.58	3 234	3.17
IV	126 174	1 198	0.95	2 887	2.29	3 507	2.78
V	64 602	560	0.87	1 387	2.15	1 705	2.64
							
*Total*	*804 854*	*8 255*	*1.03*	*19 399*	*2.40*	*25 427*	*3.16*
P**			<0.001		<0.001		<0.001

* Low apgar, neonatal distress and/or abruptio placentae

**Chi-square test for linearity exluding the unclassified category

In a multivariate analysis the odds ratio for unspecified CP of low SES compared to the highest SES category was 1.49 after adjustment for demographic confounders (Table 3; Model 2). The OR of low SES decreased to 1.36 in the presence of all three perinatal variables in the final model(Table 4; Model 6), while moderate SES decreased from OR 1.30 in Model 2 to 1.21 in Model 6 compared with high SES. Preterm birth alone accounted for 65% and 67% respectively of this decrease (Table 4; Models 3 and 6).

**Table 4:** Logistic regression of perinatal indicators and unspecified cerebral palsy.

		Model 1OR (95% C.I.)	Model 2* OR (95% C.I.)	Model 3* OR (95% C.I.)	Model 4* OR (95% C.I.)	Model 5* OR (95% C.I.)	Model 6* OR (95% C.I.)
SES							
	Unclassified	1.40 (1.08–1.80)	1.41 (1.09–1.82)	1.31 (1.01–1.69)	1.34 (1.04–1.73)	1.38 (1.07–1.78)	1.25 (0.96–1.62)
	I+II	1.51 (1.18–1.93)	1.49 (1.16–1.91)	1.40 (1.09–1.80)	1.43 (1.11–1.83)	1.46 (1.14–1.88)	1.36 (1.05–1.75)
	III+IV	1.28 (0.99–1.65)	1.30 (1.01–1.68)	1.24 (0.96–1.60)	1.24 (0.96–1.61)	1.28 (0.99–1.66)	1.21 (0.94–1.57)
	V	1	1	1	1	1	1
Gest. age							
	22–32			32.53 (28.4–37.3)			13.6 (11.5–16.1)
	33–36			3.05 (2.54–3.67)			2.28 (1.89–2.76)
	37–41			1			1
	42+			1.70 (1.05–2.76)			1.54 (0.95–2.509
Asphyxia	yes				16.5 (14.6–18.7)		7.21 (6.22–8.36)
SGA	yes					3.66 (3.05–4.38)	1.39 (1.15–1.69)

*Model is adjusted for year of birth and sex of the child, maternal age at the birth of the child and geographical residency.

## Discussion

This study of a national cohort in Sweden demonstrates a considerable socio-economic gradient for cerebral palsy. Children in households with a low SES had a 50% higher risk than those in the highest SES category of being admitted to a hospital with a diagnosis of CP. Perinatal risk factors, particularly extreme preterm birth and asphyxia were associated with unspecified CP in this study. These indicators had a significant linear association with SES and seemed to mediate approximately 25% of the increased odds ratios associated with low SES.

The single most important perinatal risk factor in this study, accounting for two thirds of the variation associated with perinatal risk in Table 4, was gestational age. Several studies have linked preterm birth with the development of bilateral spastic diplegia through periventricular haemorrhage and leukomalaciae [16]. Recent research with MRI have demonstrated that radiographic evidence of injuries in the immature brain is quite common in children with cerebral palsy born term with perinatal complications suggesting that perinatal events may easily be overestimated as causes of cerebral palsy [17]. Thus, the estimates of the perinatal risk factors in this study may be an exaggeration of the true risk of cerebral palsy associated with perinatal complications.

An exaggerated role of perinatal factors as such may also create an exaggerated role of these factors as mediators for the influence of SES on CP. This and the considerable socio-economic risk not attributable to perinatal risk factors makes it necessary to look for alternative mediating pathways for this influence. These mechanisms could include infections, nutritional factors and other mechanisms during the pregnancy that may predispose the infant to hypoxia because of placental damage [17] as well as factors related to the interaction with care in late pregnancy. The lack of a socio-economic gradient for children born preterm and small for date in this study, just as in the study of Dolk et al in the UK [5], however, seems to contradict hypotheses about diverse quality of neonatal care as a cause of socio-economic differences in cerebral palsy.

This study demonstrates a socio-economic gradient for cerebral palsy. A similar conclusion by Sundrum et al in a study population in west Sussex in the UK [3] has been challenged by Pharoah because of their inclusion of children with severe head injuries and malformation syndromes as well children with unknown SES [15]. In this study we have analysed these children separately, as suggested by Pharoah, thus giving indirect support to the conclusions made by Sundrum et al [3].

In the only other Swedish study of socio-economic differences in CP up to date, Lagergren [4] found a slightly higher incidence of CP in families with high SES compared to low SES in children born 1960–72 in an urban area of southern Sweden. These findings are in stark contrast to the socio-economic pattern in the present study. Apart from the possible importance of time and the impact of significant advances in perinatal care made during these three decades it must also be pointed out that the comparatively low number of study subjects (N = 183), the inclusion of international adoptees and the limited variation of perinatal care in a small geographical area in the Lagergren study are possible factors that may explain the discrepancy between these two studies.

### Methodological Considerations

The use of hospital discharge data to identify children with cerebral palsy is a source of possible bias and error in this study. Firstly, the quality of a hospital diagnosis of cerebral palsy has not been evaluated in Sweden. Thus, it is possible that our results were attenuated by children misclassified as having cerebral palsy when in fact they did not.

Secondly, the live birth incidence of cerebral palsy has been estimated to 2.2/1000 in cohorts born 1990–93 in southern Sweden [18] and 2.4 in western Sweden in cohorts born 1987–90 [19] suggesting that at the most 80% of the children in Sweden with CP were captured with the register design of this study. A certain bias with regards to the severity of the symptoms of CP can be anticipated, since children with more severe disabilities probably are admitted to hospitals more often than those with minor symptoms. If socio-economically disadvantaged children with CP tend to have more severe forms of CP and/or are more likely to be admitted to hospital compared to children from more advantaged backgrounds despite a similar morbidity, the magnitude of the socio-economic gradient was overestimated in this study. Further studies in more complete Swedish populations of children with cerebral palsy where the diagnosis has been confirmed by evaluated protocols are needed to confirm our findings.

Socio-economic categorisation of households with small children is not an easy task. Parents of small children are often in a transitional period on the labour market or students. Thus the validity of the socio-economic position defined by SES in the Census of 1985 in cohorts born 1987–93 is probably quite low, causing an underestimation of the "true" socio-economic gradient.

The major strength of this study is the large study population made possible by the wide coverage of the Swedish national registers. To our knowledge this is the largest cohort of children with cerebral palsy in a study of socio-economic risk factors. The identification and exclusion of children with CP related to severe head injuries and gross malformation avoided some possible pitfalls in the analysis, since these cases probably have particular risk factor profiles [15].

## Conclusion

In summary this study suggests the existence of a continuous socio-economic gradient prior to birth in families where the offspring develop CP in Sweden. Perinatal complications seem to mediate some of this gradient. Further investigations are needed to confirm our findings in more complete study populations and to identify the mechanisms that explain the considerable residual socio-economic variation.

## Competing interests

The authors declare that they have no competing interests.

## Authors' contributions

AH came up with the idea of this study inspired by KTJ, designed the study, made all analyses and wrote the first draft of the manuscript. KTJ created the theoretical framework for the analysis and participated in the interpretation of the data and the writing of the article. Both authors read and approved the final version of the article

## Pre-publication history

The pre-publication history for this paper can be accessed here:



## References

[B1] Spencer N (2000). Poverty and child health.

[B2] Hjern A (2006). Chapter 7: children's and young people's health. Scand J Public Health Suppl.

[B3] Sundrum R, Logan S, Wallace A, Spencer N (2005). Cerebral palsy and socioeconomic status: a retrospective cohort study. Arch Dis Child.

[B4] Lagergren J (1981). Children with motor handicaps. Epidemiological, medical and socio-paediatric aspects of motor handicapped children in a Swedish county. Acta Paediatr Scand Suppl.

[B5] Dolk H, Pattenden S, Johnson A (2001). Cerebral palsy, low birthweight and socio-economic deprivation: inequalities in a major cause of childhood disability. Paediatric and perinatal epidemiology.

[B6] Dowding VM, Barry C (1990). Cerebral palsy: social class differences in prevalence in relation to birthweight and severity of disability. Journal of Epidemiology and Community Health.

[B7] Thorngren-Jerneck K, Herbst A (2001). Low 5-minute Apgar score: a population-based register study of 1 million term births. Obstet Gynecol.

[B8] Hagberg B, Hagberg G, Beckung E, Uvebrant P (2001). Changing panorama of cerebral palsy in Sweden. VIII. Prevalence and origin in the birth year period 1991–94. Acta Paediatr.

[B9] Centre for Epidemiology (2003). The Swedish Medical Birth Registry. A summary of content and quality.

[B10] Marsal K, Persson PH, Larsen T, Lilja H, Selbing A, Sultan B (1996). Intrauterine growth curves based on ultrasonically estimated foetal weights. Acta Paediatr.

[B11] Statistics Sweden (1982). Socio-economic classification (SEI).

[B12] Centre for Epidemiology (2004). National Patient Discharge Register.

[B13] WHO (1987). International Classification of Diseases 9th revised edition.

[B14] WHO (1997). International Classification of Diseases 10th revised edition.

[B15] Pharoah PO (2005). Cerebral palsy: does SES make a difference?. Arch Dis Child.

[B16] Krageloh-Mann I, Petersen D, Hagberg G, Vollmer B, Hagberg B, Michaelis R (1995). Bilateral spastic cerebral palsy – MRI pathology and origin. Analysis from a representative series of 56 cases. Developmental Medicine and Child Neurology.

[B17] Bax M, Tydeman C, Flodmark O Clinical and MRI correlates of cerebral palsy: the European Cerebral Palsy Study. Jama.

[B18] Nordmark E, Hagglund G, Lagergren J (2001). Cerebral palsy in southern Sweden I. Prevalence and clinical features. Acta Paediatr.

[B19] Hagberg B, Hagberg G, Olow I, von Wendt L (1996). The changing panorama of cerebral palsy in Sweden. VII. Prevalence and origin in the birth year period 1987–90. Acta Paediatr.

